# Supplementary material for: Applicability of a Chemiluminescence Immunoassay to Screen Postmortem Bile Specimens and Its Agreement with Confirmation Analysis
Source: Int J Mol Sci. 2024 Mar 29;25(7):3825. doi: 10.3390/ijms25073825 (PMC11011420; doi:10.3390/ijms25073825)
Supplement: Supplementary file 1 [file ijms-25-03825-s001.zip › ijms-2912667-supplementary.pdf]

Table S1. Mass spectrometer compound-dependent parameters. Compound_1 quantifier. Compound_2 qualifier.

POSITIVE IONIZATION MODE							
**Analyte or IS**	**Precursor ion (m/z)**	**Product ion (m/z)**	**Retention time (min)**	**DP (V)**	**EP (V)**	**CE (V)**	**CXP (V)**
Amphetamine_1	137.1	92.0	3.4	30	10	21	10
Amphetamine_2	137.1	120.1	3.41	30	10	11	10
Amphetamine-D5	142.1	93.0	3.36	30	10	21	10
BDB_1	194.1	147.1	5.02	30	10	18	10
BDB_2	194.1	177.1	5.02	30	10	11	10
BDB-D2	196.1	149.1	5	30	10	18	10
Butylone_1	223.1	175.1	5.23	30	10	23	10
Butylone_2	223.1	130.1	5.22	30	10	59	10
Butylone-D2	225.1	177.1	5.21	30	10	27	10
2C-B_1	261.1	244.1	5.95	20	10	17	10
2C-B_2	261.1	228.1	5.95	20	10	29	10
2C-B-D5	266.1	249.1	5.88	20	10	17	10
2C-I_1	309.2	292.1	6.62	20	10	19	10
2C-I_2	309.2	277.1	6.61	20	10	33	10
2C-I-D5	314.2	297.1	6.56	20	10	19	10
Cathinone_1	150.1	105.1	3.08	30	10	25	10
Cathinone_2	150.1	117.1	3.07	30	10	31	10
Cathinone-D5	155.1	110.1	3.01	30	10	25	10
MBDB_1	209.1	136.1	5.55	25	10	27	10
MBDB_2	209.1	178.1	5.55	25	10	15	10
MBDB-D4	213.1	136.1	5.51	25	10	27	10
MDA_1	180.1	133.1	4.12	40	10	25	10
MDA_2	181.1	164.1	4.14	40	10	15	10
MDA-D4	185.1	138.1	4.09	40	10	25	10
MDEA_1	209.1	164.1	5.25	40	10	19	10
MDEA_2	209.1	106.1	5.25	40	10	39	10
MDEA-D4	213.1	163.1	5.22	40	10	19	10
MDMA_1	195.1	164.1	4.7	30	10	17	10
MDMA_2	195.1	106.1	4.7	30	10	33	10
MDMA-D2	197.1	163.1	4.68	30	10	17	10
MDPV_1	277.1	127.1	6.86	25	10	35	10
MDPV_2	277.1	176.1	6.85	25	10	31	10
MDPV-D7	284.1	134.1	6.82	25	10	37	10
Mephedrone_1	179.1	161.1	5.05	30	10	17	10
Mephedrone_2	179.1	145.1	5.05	30	10	29	10
Mephedrone-D3	181.1	163.1	5.04	30	10	17	10
Methamphetamine_1	151.1	92.0	4.08	40	10	25	10
Methamphetamine_2	151.1	120.1	4.08	40	10	15	10
Methamphetamine-D4	155.1	92.0	4.05	40	10	25	10
Methaqualone_1	252.1	133.1	7.79	110	10	37	10
Methaqualone_2	252.1	66.0	7.79	110	10	79	10
Methaqualone-D6	258.1	139.1	7.74	110	10	39	10
Methylone_1	209.1	161.1	4.41	50	10	25	10
Methylone_2	209.1	133.1	4.41	50	10	37	10
Methylone-D2	211.1	163.1	4.4	50	10	25	10
Methylphenidate_1	234.1	85.0	6.19	46	10	25	10
Methylphenidate_2	234.1	56.0	6.19	130	10	48	10
Methylphenidate-D10	244.1	93.0	6.13	130	10	48	10

PMA_1	167.1	122.1	4.21	20	10	25	10
PMA_2	167.1	150.1	4.21	20	10	13	10
Ritalinic-acid_1	221.1	85.0	5	40	10	25	10
Ritalinic-acid_2	220.1	56.0	5	40	10	61	10
Ritalinic-acid-D4	224.1	88.0	4.97	40	10	25	10
Alprazolam_1	310.2	282.1	8.69	80	10	37	10
Alprazolam_2	310.2	206.1	8.69	80	10	57	10
Alprazolam-D6	316.2	288.1	8.66	80	10	37	10
7-NH2-Clonazepam_1	286.1	222.1	5.03	105	10	35	10
7-NH2-Clonazepam_2	286.1	121.1	5.04	105	10	39	10
7-NH2-Clonazepam-D4	290.1	226.1	4.99	105	10	35	10
7-NH2-Flunitrazepam_1	285.1	136.1	5.93	100	10	37	10
7-NH2-Flunitrazepam_2	284.1	227.1	5.93	100	10	33	10
7-NH2-Flunitrazepam-D3	287.1	138.1	5.89	100	10	37	10
7-NH2-Nitrazepam_1	252.1	122.1	4.19	85	10	36	10
7-NH2-Nitrazepam_2	252.1	146.1	4.2	85	10	38	10
7-NH2-Nitrazepam-D5	257.1	121.1	4.15	85	10	36	10
Bromazepam_1	316.1	209.1	7.22	100	10	35	10
Bromazepam_2	316.1	182.1	7.22	100	10	43	10
Bromazepam-D4	320.1	213.1	7.18	100	10	35	10
Brotizolam_1	393.2	282.1	8.98	100	10	43	10
Brotizolam_2	394.2	315.1	8.98	100	10	33	10
Chlordiazepoxide_1	301.2	283.1	7.35	50	10	33	10
Chlordiazepoxide_2	300.2	247.1	7.35	50	10	45	10
Chlordiazepoxide-D5	305.2	286.1	7.31	50	10	31	10
Clobazam_1	302.2	260.1	8.29	120	10	29	10
Clobazam_2	301.2	224.1	8.29	120	10	45	10
Clobazam-D8	309.2	267.1	8.25	120	10	31	10
Clonazepam_1	316.2	270.1	7.71	70	10	35	10
Clonazepam_2	316.2	214.1	7.71	70	10	52	10
Clonazepam-D4	320.2	274.1	7.68	70	10	35	10
Demoxepam_1	287.1	180.1	7.43	110	10	31	10
Demoxepam_2	287.1	269.1	7.43	110	10	39	10
Demoxepam-D5	292.1	180.1	7.4	110	10	33	10
DA-Flurazepam_1	289.1	140.1	7.92	110	10	41	10
DA-Flurazepam_2	289.1	226.1	7.92	110	10	39	10
DA-Flurazepam-D4	293.1	140.1	7.89	110	10	43	10
DM-Flunitrazepam_1	300.2	254.1	7.41	30	10	35	10
DM-Flunitrazepam_2	300.2	198.1	7.41	30	10	51	10
DM-Flunitrazepam-D4	304.2	258.1	7.38	30	10	35	10
Diazepam_1	286.1	155.1	9.09	100	10	35	10
Diazepam_2	286.1	194.1	9.08	100	10	43	10
Diazepam-D4	290.1	154.1	9.06	100	10	37	10

Estazolam_1	296.1	268.1	8.54	75	10	33	10
Estazolam_2	296.1	206.1	8.54	75	10	57	10
Estazolam-D4	300.1	272.1	8.51	75	10	47	10
Flunitrazepam_1	315.2	269.1	8.3	80	10	35	10
Flunitrazepam_2	314.2	239.1	8.3	80	10	45	10
Flunitrazepam-D3	317.2	271.1	8.28	80	10	35	10
Flurazepam_1	388.2	315.1	8.18	90	10	35	10
Flurazepam_2	388.2	288.1	8.18	90	10	35	10
Flurazepam-D10	398.2	315.1	8.15	90	10	33	10
OH-Alprazolam_1	325.2	297.1	8.13	110	10	37	10
OH-Alprazolam_2	325.2	216.1	8.14	110	10	53	10
OH-Alprazolam-D5	330.2	302.1	8.1	110	10	37	10
OH-Midazolam_1	342.2	203.1	8.08	70	10	35	10
OH-Midazolam_2	342.2	168.1	8.08	70	10	51	10
OH-Midazolam-D4	346.2	328.1	8.05	70	10	31	10
OH-Triazolam_1	359.2	331.1	7.97	105	10	39	10
OH-Triazolam_2	359.2	176.1	7.97	105	10	37	10
OH-Triazolam-D6	365.2	337.1	7.93	105	10	39	10
OH-Bromazepam_1	332.2	303.1	6.37	80	10	27	10
OH-Bromazepam_2	332.2	315.1	6.38	80	10	21	10
OH-Bromazepam-D4	336.2	307.1	6.34	80	10	28	10
Lorazepam_1	321.2	229.1	7.58	60	10	42	10
Lorazepam_2	321.2	303.1	7.58	60	10	21	10
Lorazepam-D4	325.2	233.1	7.55	60	10	42	10
Lormetazepam_1	336.2	290.1	8.42	50	10	35	10
Lormetazepam_2	335.2	177.1	8.41	50	10	53	10
Lormetazepam-D8	342.2	296.1	8.4	50	10	35	10
Medazepam_1	271.1	242.1	8.94	60	10	27	10
Medazepam_2	272.1	208.1	8.94	60	10	39	10
Medazepam-D4	275.1	246.1	8.92	60	10	27	10
Midazolam_1	327.2	292.1	8.32	130	10	39	10
Midazolam_2	326.2	249.1	8.32	130	10	51	10
Midazolam-D6	332.2	297.1	8.29	130	10	39	10
Nitrazepam_1	282.1	236.1	7.69	100	10	33	10
Nitrazepam_2	282.1	180.1	7.68	100	10	51	10
Nitrazepam-D5	287.1	185.1	7.65	100	10	49	10
Norclobazam_1	287.1	245.1	7.57	70	10	29	10
Norclobazam_2	287.1	210.1	7.57	70	10	43	10
Norclobazam-D6	293.1	251.1	7.57	70	10	31	10
Nordiazepam_1	271.1	140.1	8.28	90	10	37	10
Nordiazepam_2	271.1	165.1	8.28	90	10	38	10
Nordiazepam-D5	276.1	140.1	8.24	90	10	37	10
Oxazepam_1	287.1	163.1	7.78	80	10	49	10
Oxazepam_2	287.1	104.1	7.78	80	10	45	10
Oxazepam-D5	292.1	163.1	7.75	80	10	49	10
Prazepam_1	326.2	272.1	9.74	60	10	31	10

Prazepam_2	326.2	140.1	9.74	60	10	49	10
Prazepam-D4	330.2	276.1	9.72	60	10	47	10
Temazepam_1	302.2	256.1	8.59	20	10	31	10
Temazepam_2	301.2	177.1	8.58	20	10	51	10
Temazepam-D6	308.2	262.1	8.56	20	10	29	10
Triazolam_1	343.2	308.1	8.53	140	10	37	10
Triazolam_2	343.2	315.1	8.53	140	10	39	10
Triazolam-D6	349.2	312.1	8.51	140	10	39	10
Gabapentin_1	172.1	137.1	3.47	100	10	23	10
Gabapentin_2	172.1	154.1	3.47	100	10	19	10
Gabapentin-D4	176.1	139.1	3.44	100	10	23	10
Pregebalin_1	160.1	55.0	2.79	100	10	31	10
Pregebalin_2	160.1	83.0	2.79	100	10	21	10
Pregebalin-D4	164.1	57.0	2.75	100	10	29	10
Promethazine_1	286.1	87.0	8.6	55	10	25	10
Promethazine_2	286.1	199.1	8.6	55	10	43	10
Promethazine-D5	291.1	92.0	8.57	55	10	21	10
Quetiapine_1	385.2	254.1	8.4	80	10	30	10
Quetiapine_2	385.2	222.1	8.4	80	10	49	10
Quetiapine-D3	388.2	255.1	8.4	80	10	30	10
Benzoylecgonine_1	291.1	169.1	5.15	45	10	24	10
Benzoylecgonine_2	290.1	82.0	5.15	45	10	32	10
Benzoylecgonine-D3	293.1	171.1	5.14	45	10	24	10
Cocaethylene_1	319.2	197.1	7.31	70	10	27	10
Cocaethylene_2	318.2	82.0	7.31	70	10	37	10
Cocaethylene-D2	321.2	199.1	7.3	70	10	27	10
Cocaine_1	305.2	183.1	6.65	40	10	26	10
Cocaine_2	304.2	77.0	6.66	40	10	75	10
Cocaine-D2	307.2	185.1	6.65	56	10	26	10
Norcocaine_1	291.1	169.1	6.77	50	10	21	10
Norcocaine_2	290.1	105.1	6.77	50	10	44	10
Norcocaine-D2	293.1	171.1	6.75	50	10	23	10
THC-COOH_1	345.2	299.1	9.84	40	10	27	10
THC-COOH_2	345.2	193.1	9.84	40	10	35	10
THC-COOH-D3	348.2	302.1	9.83	40	10	27	10
Acetylcodeine_1	342.2	225.1	6.51	130	10	35	10
Acetylcodeine_2	342.2	165.1	6.51	130	10	65	10
Acetylcodeine-D3	345.2	225.1	6.5	130	10	37	10
Buprenorphine_1	468.2	396.1	7.8	120	10	53	10
Buprenorphine_2	468.2	414.1	7.79	120	10	46	10
Buprenorphine-D4	472.2	400.1	7.77	120	10	53	10
Codeine_1	300.2	215.1	4.29	110	10	35	10
Codeine_2	300.2	165.1	4.29	110	10	55	10
Codeine-D6	306.2	218.1	4.25	110	10	35	10
Dihydrocodeine_1	302.2	199.1	4.22	120	10	45	10
Dihydrocodeine_2	302.2	128.1	4.22	120	10	79	10

Dihydrocodeine-D6	308.2	202.1	4.17	120	10	43	10
EDDP_1	279.1	234.1	9	40	10	43	10
EDDP_2	279.1	250.1	9.01	40	10	33	10
EDDP-D3	281.1	234.1	8.99	40	10	43	10
Fentanyl_1	337.2	188.1	7.87	80	10	31	10
Fentanyl_2	337.2	105.1	7.87	80	10	52	10
Fentanyl-D5	342.2	188.1	7.83	80	10	31	10
Hydrocodone_1	300.2	199.1	4.77	160	10	39	10
Hydrocodone_2	300.2	171.1	4.77	160	10	51	10
Hydrocodone-D6	306.2	202.1	4.73	160	10	41	10
Hydromorphone_1	286.1	185.1	3.15	120	10	39	10
Hydromorphone_2	286.1	157.1	3.14	120	10	53	10
Hydromorphone-D3	289.1	185.1	3.11	120	10	41	10
Meconin_1	195.1	105.1	5.71	50	10	43	10
Meconin_2	195.1	77.0	5.71	50	10	48	10
Meconin-D3	198.1	105.1	5.69	50	10	40	10
Meperidine_1	248.1	174.1	6.36	90	10	27	10
Meperidine_2	248.1	70.0	6.36	90	10	35	10
Meperidine-D4	252.1	178.1	6.34	90	10	29	10
Methadone_1	311.2	265.1	9.49	40	10	21	10
Methadone_2	311.2	106.1	9.49	40	10	33	10
Methadone-D8	319.2	268.1	9.46	40	10	21	10
6-MAM_1	328.2	165.1	4.28	130	10	52	10
6-MAM_2	328.2	211.1	4.28	130	10	35	10
6-MAM-D6	334.2	165.1	4.26	130	10	52	10
Morphine_1	286.1	165.1	2.66	120	10	52	10
Morphine_2	286.1	201.1	2.66	120	10	35	10
Morphine-D3	289.1	165.1	2.63	120	10	52	10
Naloxone_1	328.2	310.1	4.03	110	10	27	10
Naloxone_2	328.2	212.1	4.03	110	10	53	10
Naloxone-D5	333.2	315.1	3.99	110	10	27	10
Naltrexone_1	342.2	270.1	4.67	70	10	37	10
Naltrexone_2	342.2	267.1	4.67	70	10	37	10
Naltrexone-D3	345.2	270.1	4.64	70	10	39	10
Norbuprenorphine_1	414.2	83.0	6.85	120	10	73	10
Norbuprenorphine_2	414.2	187.1	6.85	120	10	48	10
Norbuprenorphine-D3	417.2	83.0	6.84	120	10	73	10
Norcodeine_1	286.1	165.1	3.59	120	10	57	10
Norcodeine_2	286.1	152.1	3.59	120	10	81	10
Norcodeine-D3	289.1	152.1	3.55	120	10	83	10
Norfentanyl_1	233.1	84.0	5.69	55	10	23	10
Norfentanyl_2	233.1	55.0	5.69	55	10	53	10
Norfentanyl-D5	238.1	84.0	5.65	55	10	25	10
Normeperidine_1	235.1	161.1	6.3	70	10	23	10

Normeperidine_2	234.1	188.1	6.3	70	10	19	10
Normeperidine-D4	238.1	164.1	6.28	70	10	23	10
Nortapentadol_1	209.1	108.1	5.34	62	10	30	10
Nortapentadol_2	208.1	121.1	5.35	28	10	62	10
Nortilidine_1	261.1	155.1	6.46	30	10	24	10
Nortilidine_2	261.1	230.1	6.46	30	10	15	10
Nortilidine-D2	263.1	155.1	6.45	30	10	24	10
O-DM-Tramadol_1	250.1	58.0	4.23	120	10	47	10
O-DM-Tramadol_2	250.1	42.0	4.23	120	10	105	10
O-DM-Tramadol-D6	256.1	64.0	4.21	120	10	49	10
Oxycodone_1	316.2	298.1	4.58	90	10	27	10
Oxycodone_2	316.2	241.1	4.58	90	10	39	10
Oxycodone-D6	322.2	304.1	4.53	90	10	27	10
Oxymorphone_1	302.2	227.1	2.78	110	10	37	10
Oxymorphone_2	302.2	284.1	2.78	110	10	27	10
Oxymorphone-D3	305.2	230.1	2.74	110	10	37	10
Papaverine_1	341.2	203.1	8.24	114	10	36	10
Papaverine_2	340.2	296.1	8.24	114	10	47	10
Papaverine-D3	343.2	205.1	8.21	114	10	40	10
Propoxyphene_1	341.2	58.0	8.34	50	10	49	10
Propoxyphene_2	341.2	267.1	8.34	50	10	13	10
Propoxyphene-D4	345.2	58.0	8.32	50	10	13	10
Sufentanil_1	388.2	238.1	8.57	70	10	27	10
Sufentanil_2	388.2	112.1	8.56	70	10	47	10
Sufentanil-D4	392.2	238.1	8.54	70	10	27	10
Tapentadol_1	223.1	108.1	5.45	20	10	33	10
Tapentadol_2	222.1	121.1	5.45	20	10	29	10
Tapentadol-D3	225.1	107.1	5.45	20	10	33	10
Thebaine_1	312.2	58.0	7.36	30	10	45	10
Thebaine_2	312.2	251.1	7.36	30	10	41	10
Thebaine-D3	315.2	61.0	7.35	30	10	45	10
Tilidine_1	275.1	155.1	6.98	40	10	27	10
Tilidine_2	275.1	78.0	6.98	40	10	63	10
Tilidine-D5	280.1	155.1	6.96	40	10	31	10
Tramadol_1	265.1	58.1	5.88	40	10	45	10
Tramadol_2	264.1	42.1	5.88	40	10	105	10
Tramadol-D3	267.1	58.1	5.86	40	10	51	10
Zaleplon_1	307.2	237.1	8.22	100	10	37	10
Zaleplon_2	307.2	234.1	8.22	100	10	53	10
Zaleplon-D3	311.2	237.1	8.19	100	10	40	10
Zolpidem_1	309.2	235.1	7.6	25	10	47	10
Zolpidem_2	309.2	264.1	7.6	25	10	37	10
Zolpidem-D5	314.2	235.1	7.55	25	10	53	10
Zopiclone_1	390.2	246.1	6.58	30	10	23	10
Zopiclone_2	390.2	218.1	6.59	30	10	46	10
Zopiclone-D3	393.2	245.1	6.57	30	10	23	10

Ketamine_1	239.1	126.1	6.23	40	10	33	10
Ketamine_2	238.1	220.1	6.23	40	10	20	10
Ketamine-D4	242.1	129.1	6.19	40	10	33	10
LSD_1	324.2	223.1	7.06	25	10	33	10
LSD_2	324.2	208.1	7.07	25	10	41	10
LSD-D3	327.2	226.1	7.05	25	10	33	10
Mescaline_1	212.1	165.1	3.43	20	10	31	10
Mescaline_2	212.1	180.1	3.43	20	10	25	10
Mescaline-D9	221.1	171.1	3.34	20	10	31	10
Norketamine_1	225.1	126.1	5.63	50	10	35	10
Norketamine_2	225.1	208.1	5.63	50	10	17	10
Norketamine-D3	228.1	129.1	5.58	50	10	33	10
O-H-LSD_1	356.2	222.1	4.82	60	10	45	10
O-H-LSD_2	356.2	166.1	4.82	60	10	71	10
PCP_1	244.1	86.0	8.41	40	10	17	10
PCP_2	244.1	91.0	8.41	40	10	37	10
PCP-D5	249.1	86.0	8.38	40	10	17	10
**NEGATIVE IONIZATION MODE**							
**Analyte or IS**	**Precursor ion (m/z)**	**Product ion (m/z)**	**Retention time (min)**	**DP (V)**	**EP (V)**	**CE (V)**	**CXP (V)**
Allobarbital_1	207.0	42.0	4.08	-50	-10	-40	-10
Allobarbital_2	207.0	84.9	4.08	-50	-10	-17	-10
Amobarbital_1	225.0	42.0	5.91	-50	-10	-45	-10
Amobarbital_2	225.0	181.9	5.91	-50	-10	-18	-10
Barbital_1	183.0	42.0	2.83	-50	-10	-40	-10
Barbital_2	183.0	139.9	2.83	-50	-10	-17	-10
Butalbital_1	223.0	42.0	5.26	-50	-10	-45	-10
Butalbital_2	223.0	179.9	5.26	-50	-10	-17	-10
Butalbital-D5	228.0	42.0	5.23	-50	-10	-40	-10
Hexobarbital_1	234.9	42.0	6.28	-45	-10	-45	-10
Pentobarbital_1	225.0	42.0	6.01	-55	-10	-40	-10
Pentobarbital_2	225.0	181.9	6.01	-55	-10	-17	-10
Pentobarbital-D5	230	42.0	5.99	-55	-10	-40	-10
Phenobarbital_1	230.9	42.0	5.05	-35	-10	-40	-10
Phenobarbital_2	230.9	187.9	5.05	-35	-10	-15	-10
Phenobarbital-D5	235.9	42.0	5.01	-35	-10	-40	-10
Secbutabarbital_1	211	42.0	5.09	-40	-10	-40	-10
Secbutabarbital_2	211	167.9	5.09	-40	-10	-20	-10
Secobarbital_1	236.9	42	6.37	-40	-10	-40	-10
Secobarbital_2	236.9	193.9	6.37	-40	-10	-17	-10
Secobarbital-D5	241.9	42.0	6.37	-40	-10	-40	-10
Thiopental_1	240.9	58.0	6.74	-35	-10	-45	-10
Thiopental_2	240.9	100.9	6.74	-35	-10	-20	-10

Table S2. Evaluation of sensitivity.

Compound	Limit of quantification (µg/L)	Limit of detection (µg/L)
Amphetamine	10.85	2.17
BDB	26.7	2.17
Butylone	11.05	0.442
2C-B	23.9	1.93
2C-I	10.45	2.09
Cathinone	11,175	2,235
MBDB	10.65	0.426
MDA	25.2	0.212
MDEA	26.7	0.421
MDMA	25.8	0.396
MDPV	27.1	2,145
Mephedrone	26.8	2,095
Methamphetamine	4.33	0.433
Methaqualone	27.6	2.12
Methylone	3.91	1,955
Methylphenidate	27.5	2.18
PMA	27.3	2.1
Ritalinic acid	25.5	2,015
Alprazolam	26.1	0.21
7-Aminoclonazepam	9,425	0.1885
7-Aminoflunitrazepam	9,225	1,845
7-Aminonitrazepam	29.6	0.251
Bromazepam	26.9	2,115
Brotizolam	27.9	2,235
Chlordiazepoxide	8.5	8.5
Clobazam	28	0.2105
Clonazepam	25.6	0.404
Demoxepam	2.16	0.432
Desalkylflurazepam	27.2	0.2125
Desmethylflunitrazepam	28.9	0.227
Diazepam	10,725	4.29
Estazolam	29.9	2.34
Flunitrazepam	26.2	4.15
Flurazepam	26.9	2.06
α-Hydroxyalprazolam	25.4	2,045
α-Hydroxymidazolam	4.24	0.212
α-Hydroxytriazolam	10.3	2.06
3-Hydroxybromazepam	25.5	2,035
Lorazepam	10,325	2,065
Lormetazepam	26.7	2.22
Medazepam	2,235	0.447
Midazolam	25.2	0.393
Nitrazepam	21.7	0.185

Norclobazam	28.7	0.449
Nordiazepam	27.2	0.224
Oxazepam	4.13	2,065
Prazepam	28.8	0.228
Temazepam	25.4	2.09
Triazolam	10,775	0.2155
Gabapentin	10,475	2,095
Pregabalin	10.25	0.205
Promethazine	10.5	2.1
Quetiapine	27.2	0.214
Benzoylecgonine	15.5	1.24
Cocaethylene	16.9	0.267
Cocaine	16.9	0.133
Norcocaine	15.5	1,215
11-nor-9-Carboxy-Δ9-THC	6.96	2.65
Acetylcodeine	10,975	0.022
Buprenorphine	0.843	0.0084
Codeine	4,775	0.0955
Dihydrocodeine	12.2	0.0975
EDDP	26	2.04
Fentanyl	5.07	0.0392
Hydrocodone	5.6	0.112
Hydromorphone	5.49	0.0438
Meconin	9,775	1,955
Meperidine	13.3	0.216
Methadone	28.5	4.55
6-Monoacetylmorphine	2.68	0.2155
Morphine	1,065	0.213
Naloxone	28.2	0.2235
Naltrexone	28	0.434
Norbuprenorphine	0.594	0.594
Norcodeine	5,675	1,135
Norfentanyl	5.81	0.0907
Normeperidine	15.1	5,825
Nortapentadol	6.05	1.21
Nortilidine	27.7	2,175
O-Desmethyltramadol	4.27	0.2135
Oxycodone	2,125	0.2125
Oxymorphone	2.1	0.105
Papaverine	28.3	2,275
Propoxyphene	28.3	0.436
Sufentanil	27.8	0.437
Tapentadol	12.9	2.03
Thebaine	25.6	0.408
Tilidine	26.3	0.42
Tramadol	2,095	0.2095

Zaleplon	24.6	0.2045
Zolpidem	11,175	0.447
Zopiclone	10,525	0.421
Ketamine	10,675	0.427
LSD	0.477	0.0372
Mescaline	5.15	0.884
Norketamine	4.54	0.454
2-Oxo-3-hydroxy-LSD	0.2125	0.0043
PCP	5.58	0.0863
Allobarbital	79.6	12.4
Amobarbital	81.5	81.5
Barbital	73.7	31.25
Butalbital	77.8	30.75
Hexobarbital	84.6	84.6
Pentobarbital	67.4	67.4
Phenobarbital	77	31
Secbutabarbital	80	31
Secobarbital	74.3	74.3
Thiopental	74.2	1.15

Table S3. Evaluation of matrix effect, recovery and efficiency of the entire process.

Compound	Matrix effect (%)			Recovery (%)			Process efficiency (%)		
	QCI	QCII	QCIII	QCI	QCII	QCIII	QCI	QCII	QCIII
Amphetamine	99.52	100.02	95.24	104.50	107.87	107.67	104.00	107.89	102.55
BDB	111.32	92.63	95.15	97.78	106.93	110.20	108.85	99.05	104.85
Butylone	91.76	95.03	92.62	118.79	107.36	110.42	109.00	102.02	102.27
2C-B	97.72	100.44	96.94	104.38	101.92	105.24	101.99	102.36	102.02
2C-I	103.18	99.18	94.04	106.75	105.85	108.17	110.15	104.98	101.72
Cathinone	97.70	100.51	97.05	108.15	106.95	111.26	105.65	107.49	107.98
MBDB	103.75	91.00	94.68	102.47	112.90	107.27	106.31	102.74	101.56
MDA	92.99	98.32	98.13	113.98	106.11	112.00	105.99	104.33	109.90
MDEA	91.83	93.54	86.93	103.23	111.38	112.64	94.79	104.19	97.91
MDMA	99.58	94.54	89.18	102.25	102.64	114.73	101.83	97.03	102.32
MDPV	111.52	96.82	97.66	97.59	110.88	106.46	108.82	107.35	103.97
Mephedrone	97.65	100.83	90.35	109.74	102.16	113.88	107.16	103.01	102.88
Methamphetamine	97.98	97.86	95.49	110.27	111.95	110.45	108.05	109.56	105.47
Methaqualone	70.05	75.45	70.79	97.52	100.14	111.22	68.32	75.55	78.73
Methylone	96.38	93.92	89.82	114.64	107.27	111.39	110.49	100.75	100.05
Methylphenidate	95.30	95.59	89.68	108.58	108.94	101.52	103.47	104.13	91.04
PMA	89.10	91.29	90.34	122.40	111.20	116.10	109.06	101.51	104.89
Ritalinic-acid	99.53	98.16	86.59	88.54	111.91	124.42	88.13	109.86	107.73
Alprazolam	87.25	73.77	97.39	104.04	115.28	93.82	90.78	85.04	91.38
7-NH2-Clonazepam	93.71	93.65	96.63	106.33	99.35	98.05	99.64	93.04	94.75
7-NH2-Flunitrazepam	101.34	100.76	101.36	97.30	111.61	97.93	98.61	112.46	99.26

7-NH2-Nitrazepam	101.20	98.64	84.93	79.09	79.00	88.37	80.04	77.92	75.05
Bromazepam	95.51	92.19	92.18	103.14	117.39	112.73	98.51	108.22	103.91
Brotizolam	91.32	89.78	91.65	137.87	142.04	144.71	125.90	127.53	132.62
Chlordiazepoxide	88.17	94.96	91.36	110.88	114.06	109.74	97.77	108.31	100.26
Clobazam	91.94	89.57	91.99	122.09	108.72	116.80	112.25	97.38	107.45
Clonazepam	100.52	98.61	87.23	107.99	103.62	117.11	108.55	102.18	102.16
Demoxepam	96.71	94.09	86.95	100.59	114.41	119.28	97.28	107.65	103.71
DA-Flurazepam	102.20	102.52	98.45	112.13	118.45	110.71	114.59	121.43	109.00
DM-Flunitrazepam	91.52	94.23	91.88	111.08	104.26	114.82	101.66	98.24	105.50
Diazepam	97.90	92.37	93.40	124.31	121.64	120.89	121.70	112.36	112.92
Estazolam	97.92	91.57	94.92	110.00	110.40	114.73	107.72	101.09	108.91
Flunitrazepam	83.39	88.89	90.11	109.11	100.39	114.28	90.98	89.23	102.98
Flurazepam	95.71	95.16	92.53	104.99	107.14	112.05	100.49	101.95	103.68
OH-Alprazolam	90.67	120.45	89.76	125.52	106.44	113.75	113.81	128.21	102.10
OH-Midazolam	98.24	101.09	97.13	110.69	98.03	98.88	108.74	99.10	96.04
OH-Triazolam	76.38	84.84	81.00	106.59	105.57	103.06	81.41	89.57	83.48
OH-Bromazepam	87.22	93.72	90.35	128.60	111.50	112.45	112.17	104.50	101.60
Lorazepam	100.89	89.38	94.32	100.29	114.11	110.28	101.19	101.99	104.02
Lormetazepam	94.35	91.17	99.11	115.17	126.56	115.32	108.66	115.39	114.29
Medazepam	101.32	98.70	88.93	101.09	102.58	108.16	102.42	101.25	96.19
Midazolam	99.68	96.11	90.08	121.57	97.85	110.23	121.19	94.04	99.30
Nitrazepam	88.20	89.61	90.76	124.55	107.46	104.80	109.86	96.30	95.12
Norclobazam	100.25	95.56	93.82	110.12	106.10	111.39	110.39	101.39	104.50
Nordiazepam	101.68	96.70	91.55	82.87	91.41	87.20	84.26	88.39	79.82
Oxazepam	102.29	103.23	92.23	117.24	123.35	119.33	119.93	127.33	110.06
Prazepam	94.25	92.16	90.62	109.89	109.23	114.56	103.57	100.67	103.81
Temazepam	99.64	94.67	94.11	103.82	115.62	107.63	103.45	109.45	101.29
Triazolam	100.43	104.81	86.14	114.76	110.83	124.91	115.26	116.16	107.59
Gabapentin	100.28	96.72	92.50	104.86	104.69	111.01	105.16	101.25	102.68
Pregebalin	102.44	99.57	94.46	104.07	104.33	112.17	106.62	103.88	105.96
Promethazine	109.09	108.47	93.91	111.01	100.67	116.51	121.11	109.19	109.42
Quetiapine	101.50	94.34	89.56	114.73	104.63	111.52	116.44	98.71	99.88
Benzoylecgonine	92.05	93.71	88.65	114.27	111.08	110.29	105.18	104.10	97.77
Cocaethylene	104.47	93.86	86.46	105.98	109.19	115.59	110.72	102.49	99.94
Cocaine	85.15	97.64	91.48	101.83	106.32	105.90	86.70	103.81	96.88
Norcocaine	97.53	98.71	90.44	106.10	111.22	115.79	103.48	109.79	104.72
THC-COOH	81.73	75.96	73.32	339.42	421.61	362.67	277.40	320.24	265.89
Acetylcodeine	98.11	99.26	101.88	109.33	107.84	96.46	107.27	107.05	98.27
Buprenorphine	70.65	76.58	88.22	250.65	128.38	94.33	177.07	98.32	83.22
Codeine	100.31	108.80	85.77	107.02	105.59	104.81	107.35	114.88	89.89
Dihydrocodeine	98.46	96.79	89.53	109.41	105.38	108.42	107.73	101.99	97.07
EDDP	95.70	96.41	91.69	113.48	109.93	106.26	108.60	105.98	97.43
Fentanyl	88.14	97.69	94.59	125.95	109.01	108.67	111.01	106.49	102.80
Hydrocodone	98.55	98.85	93.18	105.96	112.49	113.45	104.42	111.19	105.71
Hydromorphone	110.64	94.74	92.95	102.96	111.65	105.82	113.92	105.78	98.36
Meconin	102.92	93.90	96.81	103.36	109.47	103.60	106.38	102.80	100.29

Meperidine	100.12	101.23	91.84	98.89	105.82	111.96	99.02	107.13	102.82
Methadone	98.48	95.08	89.09	118.41	115.13	114.35	116.61	109.46	101.88
6-MAM	79.52	94.99	96.58	103.24	116.18	104.71	82.09	110.36	101.13
Morphine	99.84	95.40	93.98	107.29	106.94	108.02	107.12	102.02	101.52
Naloxone	96.02	88.67	85.91	107.24	113.25	114.07	102.97	100.43	97.99
Naltrexone	96.75	91.43	89.00	106.86	116.32	108.87	103.39	106.35	96.89
Norbuprenorphine	259.59	74.58	89.73	101.39	171.65	100.47	263.19	128.02	90.15
Norcodeine	99.70	101.90	96.42	105.70	103.29	103.33	105.38	105.25	99.63
Norfentanyl	101.19	89.15	85.29	110.91	111.18	110.61	112.22	99.12	94.33
Normeperidine	105.07	92.90	94.38	100.84	113.35	111.76	105.95	105.30	105.48
Norpropoxyphene	113.35	92.85	99.60	137.80	171.40	136.86	156.20	159.14	136.31
Nortapentadol	94.49	86.50	87.14	107.01	110.03	108.48	101.11	95.18	94.53
Nortilidine	112.05	94.64	94.40	105.51	110.08	103.79	118.22	104.18	97.98
O-DM-Tramadol	102.53	112.32	92.29	103.81	102.87	113.49	106.44	115.55	104.75
Oxycodone	96.40	101.14	93.85	110.77	104.00	109.53	106.78	105.18	102.80
Oxymorphone	105.55	100.75	92.30	110.79	115.70	111.15	116.94	116.57	102.59
Papaverine	89.89	95.65	83.82	120.44	120.73	117.94	108.26	115.47	98.86
Propoxyphene	94.51	98.58	87.23	105.91	105.16	114.67	100.09	103.66	100.02
Sufentanil	99.79	99.46	90.49	118.69	110.27	115.50	118.43	109.68	104.51
Tapentadol	109.71	95.35	88.64	103.22	109.05	106.95	113.25	103.98	94.80
Thebaine	96.14	93.50	95.14	115.66	116.36	113.62	111.20	108.79	108.09
Tilidine	96.31	102.03	93.93	104.05	106.97	111.53	100.21	109.14	104.76
Tramadol	101.01	95.48	92.71	101.92	108.26	118.11	102.95	103.36	109.50
Zaleplon	103.69	107.57	89.67	95.52	112.93	114.98	99.04	121.48	103.11
Zolpidem	98.52	96.92	93.29	112.17	103.73	113.02	110.52	100.53	105.43
Zopiclone	90.00	90.83	94.90	117.01	106.61	105.87	105.30	96.83	100.47
Ketamine	99.61	90.80	86.10	106.61	115.71	108.04	106.19	105.06	93.03
LSD	95.03	90.28	96.23	111.25	99.48	115.77	105.72	89.81	111.40
Mescaline	90.73	89.31	87.00	122.84	104.37	123.62	111.45	93.21	107.55
Norketamine	103.50	95.76	90.85	105.17	109.93	108.57	108.85	105.27	98.64
O-H-LSD	106.83	86.62	84.27	102.42	100.64	116.33	109.41	87.18	98.04
PCP	100.80	97.88	85.59	106.97	103.24	114.50	107.83	101.05	98.00
Allobarbital	115.90	118.26	121.38	105.12	86.23	110.72	121.84	101.98	134.38
Amobarbital	82.25	95.23	97.26	123.77	103.67	102.22	101.81	98.72	99.42
Barbital	125.19	121.86	126.14	103.60	79.52	107.48	129.70	96.91	135.58
Butalbital	105.48	109.25	87.77	113.87	96.92	116.96	120.11	105.89	102.66
Hexobarbital	49.43	119.86	104.88	102.45	77.57	118.09	50.64	92.97	123.85
Pentobarbital	117.26	80.07	107.60	89.28	129.94	96.42	104.69	104.04	103.75
Phenobarbital	116.25	94.49	105.35	102.32	91.48	113.40	118.95	86.43	119.47
Secbutabarbital	113.84	112.67	98.05	99.56	96.80	113.10	113.34	109.07	110.90
Secobarbital	76.87	123.75	93.21	123.28	102.86	108.90	94.77	127.29	101.51
Thiopental	87.03	112.36	86.03	116.26	106.16	134.76	101.19	119.28	115.94
